# Attempted suicide by Melatonin overdose: Case report and literature review

**DOI:** 10.1192/j.eurpsy.2022.2166

**Published:** 2022-09-01

**Authors:** T. Gutierrez Higueras, F. Calera Cortés, A. Trives Muñoz, S. Vicent Forés, S. Sainz De La Cuesta Alonso

**Affiliations:** 1 Reina Sofia University Hospital, Psychiatry, Córdoba, Spain; 2 Hospital Reina Sofía Córdoba, Psychiatry, Cordoba, Spain; 3 Hospital Reina Sofía Córdoba, Psychiatry, Córdoba, Spain

**Keywords:** emergency, melatonine, Suicide, overdose

## Abstract

**Introduction:**

Melatonine (N-acetyl-5-methoxytryptamine) is an endogenous neurohormone produced by pineal gland. It is related to sleep-wake circadian rhythms, and nowdays it is sold without prescription as a “natural treatment” for sleep disorders. Most common side effects of melatonin overdose are drowsiness, dizziness, fatigue, headache, confusion, nightmare, hypotension, tachycardia and hypothermia. Supportive measures and control of vital signs are essential for an early discharge of the patient.

**Objectives:**

To present a case of an 42-year-old woman who was taken to the emergency department after voluntary ingestion of 60 tables of melatonin 2mg (Total amount 120mg), in a suicide attempt. To describe the most common side effects of melatonine overdose a the literature review.

**Methods:**

Clinical case presentation and retrospective literature review.

**Results:**

A 42-year-old woman who was taken to the emergency department after voluntary ingestion of 60 tables of melatonin 2mg (Total amount 120mg), about 1 hour before coming, in a suicide attempt. After clinical evalutation, gastric lavage was performed. ang 50g activated charcoal given. Drowiness and mild hypothermia (34ºC) was detected. After 12 hours of vital signs observation the patient was discharged and to psychiatry consultation, where depressive mood disorder and chronic insomnia was diagnosed.

**Conclusions:**

Melatonin is one of the least toxic medication. Most common side effects of overdose are drowsiness, dizziness, fatigue, headache, confusion, nightmare, hypotension, tachycardia and hypothermia. Supportive measures and control of vital signs are essential for the treatment.

**Disclosure:**

No significant relationships.

